# Histological and morphometrical evaluation of non-critical bone defects after treatment with biomaterial and bisphosphonates

**DOI:** 10.4317/medoral.27033

**Published:** 2025-03-23

**Authors:** Gustavo Vicentis Oliveira Fernandes, Bruno Gomes dos Santos Martins, Artak Heboyan, Rogerio Moraes Castilho, Juliana Campos Hasse Fernandes

**Affiliations:** 1 ORCID: 0000-0003-3022-4390. PhD. A. T. Still University, Missouri School of Dentistry and Oral Health, St. Louis, MO, USA; 2 Universidade Católica Portuguesa, Faculty of Dental Medicine, Center for Interdisciplinary Research in Health, Viseu, Portugal; 3 PhD student. Doctoral Program “Surgery and Dentistry” Department of Surgery - University of Salamanca, Salamanca, Spain; 4 PhD. Department of Research Analytics, Saveetha Dental College and Hospitals, Saveetha Institute of Medical and Technical Sciences, Saveetha University, Chennai, India; 5 Department of Prosthodontics, Faculty of Stomatology, Yerevan State Medical University after Mkhitar Heratsi, Yerevan, Armenia; 6 PhD. University of Michigan, School of Dentistry, Periodontics and Oral Medicine Department, Ann Arbor, MI, USA; 7 ORCID: 0000-0001-7603-3544. DDS. Independent Researcher, St. Louis, MO, USA

## Abstract

**Background:**

The primary goal of this *in vivo* study was to ascertain if systemic bisphosphonates (BPs) positively affect bone repair in non-critical defects when assisted with a carbonated hydroxyapatite graft biomaterial (Biomat).

**Material and Methods:**

Thirty-six female rats were allocated into two control groups (blood clot [BC] and alloplastic biomaterial); two groups with zoledronate (third-generation BPs): Zol.BP and Zol.BP+Biomat; and two groups with clodronate (first-generation BPs): Clod.BP and the Clod.BP+Biomat. The experimental groups started the application of BP 60 days before surgery. Then, a 2 mm non-critical defect was performed in the rats’ femur and filled according to the group. All animals were euthanized 30 days after surgery, and the samples were collected for histological and histomorphometry analysis, respectively, for descriptive and quantitative analyses.

**Results:**

Zol.BP+Biomat had greater new bone formation, whereas clodronate presented high osteogenic potential, significantly increasing the observed levels of newly formed bone even in the absence of the biomaterial. Histomorphometrically, BC had 2% bone formation compared to the biomaterial group (5%). Zol.BP and Col.BP achieved bone formation of 6-fold (12%, *p*>0.05) and 9.5-fold (19%, *p*<0.05), respectively, when compared with BC. Zol.BP+Biomat group presented the highest value found for newly formed bone (24%), 12-fold more than BC (*p*<0.001) and 4.8-fold more than the biomaterial group (*p*<0.01).

**Conclusions:**

It is possible to conclude that the systemic use of BP positively affected non-critical bone defects when associated with biomaterials, mainly when the third generation of BPs was used in this association.

** Key words:**Alloplastic biomaterial, bone defect, bone healing, in vitro, bisphosphonates.

## Introduction

A class of medications known as bisphosphonates (BPs) has demonstrated efficacy in the prevention and treatment of bone diseases (hypercalcemia, osteolytic lesions of multiple myeloma, pathological fractures, osteoporosis, osteopenia, and bone metastases linked to soft tissue tumors like lung, prostate, or breast cancer). BPs prevent bone resorption, reduce pain, and prevent additional complications (1-4) by directly or indirectly affecting osteoclasts, which undergo either apoptosis or lose their capacity to differentiate from hematopoietic stem cells (3,5). BPs are classified according to generations. The first generation is considered a non-nitrogenous and includes Clodronate, Etidronate, and Tiludronate, whereas the second (Alendronate, Neridronate, and Pamidronate) and third generation (Risedronate, Minodronate, Zoledronate, and Ibandronate) contain nitrogen in their formula (6).

Nitrogenated BPs and non-nitrogenous differ in their mechanisms of action. After being taken up by osteoclasts during the process of bone resorption, nitrogenated BPs block the mevalonate pathway, which stops proteins from being prenylated. Prenylation is crucial for the correct function of important intracellular proteins (1,2). Conversely, non-nitrogenous bisphosphonates resemble adenosine triphosphate (ATP) and impede the activity of mitochondria. Both processes cause the osteoclasts to activate apoptosis (1,2). Another role of BP is to stimulate osteoblast development and increase the proliferation of stromal cells derived from human bone marrow cells, both of which aid in producing new bone (7,8). Because of these characteristics, several studies have investigated how BPs, used either systemically or locally, affect bone in terms of preventing resorption and/or improving its formation (9-11).

BPs can be administrated orally or intravenously (6). The administration of intravenous BPs has shown a higher chance of developing avascular bone necrosis in the jaw, known as medication-related osteonecrosis of the jaw (MRONJ) or implant loss, compared to oral-intaking therapy (12). Patients who received intravenous bisphosphonates experienced an implant failure rate of 8.82%, in contrast to a 1.18% failure rate for those undergoing intraoral therapy, a decrease by a factor of approximately 7.47 times (13). Zoledronate (with a single dose injection, 0.1 mg/kg) and Alendronate (oral administration, 7 mg/kg/week), both nitrogenous BPs, showed improvement in titanium implant osseointegration in ovariectomized rats (14).

A comparison of the effects of systemic administration of different generations of BPs on bone healing and their impact on the biological response to a grafted biomaterial has not been performed despite some studies evaluating the association of BPs with biomaterials. Thereby, the primary goal of this study was to assess whether systemically administered zoledronate (3rd generation BP) and clodronate (1st generation BP) have a positive effect on bone repair in non-critical defect when assisted by synthetic bone graft biomaterial. Secondarily, evaluate whether different generations of BPs will present different outcomes in terms of bone formation and biomaterial remnant.

## Material and Methods

This investigation was approved by the Ethics Committee for the welfare of experimental animals of the University (no. 4.1300.36). All analyses were conducted by the principles of Good Laboratory Practice and ARRIVE (Animal Research: Reporting of In Vivo Experiments) guidelines. Thirty-six female rats (Rattus norvegicus), 4-month-old females, and body weight 250 ± 20g were included and randomly allocated into six groups (according to the treatment). Animals were acclimatized and then housed under standard controlled conditions: polypropylene cages (dimension 15cm×30cm×40cm, filled with shavings of white pine), 12h light/dark cycle with the light beginning at 7:00 AM, an ambient temperature (22±2°C), and humidity (45-55%). The animals had *ad libitum* access to water and food.

- Groups and allocation

The six groups were: (A) Negative Control Group, filled only with blood clots; (B) Positive Control (Biomat) Group, received only the alloplastic biomaterial; (C) Zol.BP: the animals were treated with zoledronic acid, and only blood clots filled the defect; (D) Biomat+Zol.BP: received zoledronic acid and alloplastic biomaterial for treatment of the defect; (E) Clo.BP: the animal was treated with clodronate, and the defect was left only with blood clots; (F) Biomat+Clo.BP: received clodronate, and the defect was treated with alloplastic biomaterial (Table 1). The biomaterial was a non-sintered carbonated hydroxyapatite with low crystallinity and 450μm of diameter (stoichiometry ratio of 1.67).

- Surgical procedures and sample collection

The rats in groups C-F received bisphosphonate injections sixty days before any surgical procedure. The zoledronic acid (Novartis Pharma AG, Basel, Switzerland) was applied intraperitoneally (0.6mg/kg, every 30 days - a total of three applications), and the clodronate (Jenahexal Pharma GmbH, Thuringia, Germany) was applied intraperitoneally (20mg/kg, every 30 days - total of three application). The surgical procedures (performed 60 days after the initial application of BP) were carried out under general anesthesia using 75mg/kg of ketamine and 1.5mL/kg of Rompun, administered intramuscularly. After trichotomy of the femoral region, a linear incision was made in the skin overlying the femur, followed by total displacement of the skin and periosteum. Then, a 2mm defect was performed with a spherical carbide bur of 2mm in diameter, with a handpiece at 1200 RPM, under saline solution irrigation. After filling the defect, if necessary, the tissues were repositioned and sutured with nylon 5.0 (Ethicon®, Johnson & Johnson, U.S.A.).

After 30 days of the surgical procedure, an overdose of anesthesia was administrated with ketamine (75mg/kg) and xylazine (5mg/kg) intraperitoneally. Subsequently, the right femur was collected and fixated in 10% buffered formalin. The samples were dehydrated (successive alcohol baths [70%-100%]), decalcified, and processed for paraffin embedding; subsequently, 5μm sections were obtained and stained with hematoxylin and eosin (H&E).

- Histological and morphometrical analysis

One professional (GVOF) performed histological evaluation using a microscope (Microscope Z1 - Zeiss, Göttingen, Germany). It was assessed the pre-existing bone, new bone, medullary tissue, biomaterial, and other structures. Then, the histological description was performed for each group. In addition, the morphometrical analysis for the same parameters mentioned above was done using the software ImagePro Plus® (v. 6.0, Media Cybernetics, Silver Spring, U.S.A.).

- Statistical analysis

Statistical analysis was performed with GraphPad® Prism (v. 9.0, GraphPad Software, San Diego, CA, USA). After evaluating the normal distribution of the data obtained (Kolmogorov-Smirnov test), it was used the non-parametrical test (Kruskal-Wallis) with a post-hoc test of Dunn to detect statistically significant differences at *p* < 0.05.

## Results

**Figure 1:**
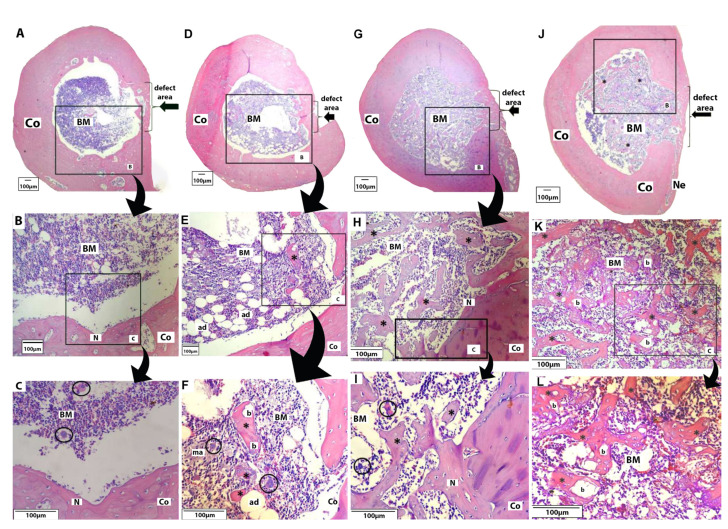
A-C) Negative Control Group [Blood clot]; A. Defect filled by new bone [lateral black arrow]; medullary cavity observed composed of cells from bone marrow [BM], surrounded by cortical bone [Co] [magnification: 4x]; B. It is possible to verify an endosteal reaction with a newly formed bone [N] [magnification: 40x]; C. New bone with the presence of osteocytes and megakaryocytes [circles] [magnification: 100x]. D-F) Positive Control [Biomat] Group; D. Defect entirely filled by new bone [lateral arrow] [magnification: 4x]; E-F. Bone marrow [BM] surrounded by cortical bone [Co] with the presence of adipocytes [ad], macrophages [ma], and megakaryocytes [circle]; islands of newly formed bone [*] involving biomaterial [b] [magnification, respectively, 40x and 100x]. G-I) Zol.BP Group; G. Defect entirely filled with new bone [lateral arrow] with bone marrow [BM] presents internally [magnification: 4x], and the medullary cavity lies surrounded by compact bone [Co]; H-I. Presence of megakaryocytes [circles], interspersed with many trabeculae of new bone [*], presence of a small portion of endosteal newly formed bone [N] [magnification, respectively, 40x and 100x]. J-L) Zol.BP + Biomat Group. J-K. Defect area filled with new bone [lateral arrow], and an external periosteal reaction was observed, originating new bone [Ne]; bone marrow [BM] with interspersed of many trabeculae of new bone [*], sometimes in close contact with the biomaterial [b] - osteoconduction property [magnification, respectively, 4x and 40x]. L. The medullary area is surrounded by cortical bone [Co], and many new trabeculae can be observed in close contact with the partially resorbed particles of the biomaterial [magnification: 100x].

There were no surgical complications during the investigation. After 90 days, the animals were healthy and showed no macroscopic evidence of infection. At 30 days following the biomaterial implantation, the histological assessments of the control group showed that the defect was entirely filled by new bone; this proves that the type of defect (2mm ø) was not critical. Additionally, there was a minimal endosteal response with new bone formation and inflammatory infiltrates in all experimental groups, specifically in the control group identified by the presence of megakaryocytes (Fig. 1).

**Figure 2:**
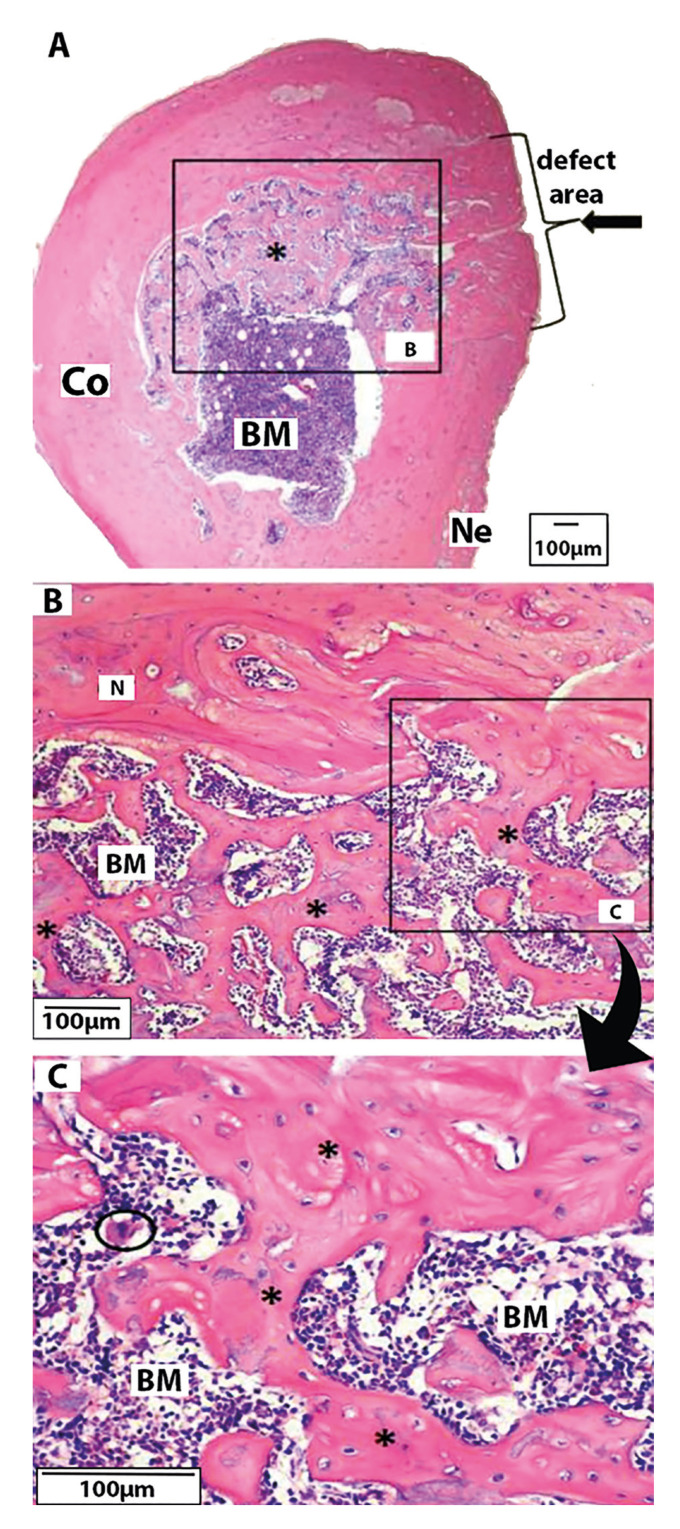
Clo.BP Group. A. Defect area entirely filled with a thick mature bone (lateral arrow); central zone with bone marrow (BM) and a dense presence of newly formed trabeculae bone (*), surrounded by cortical bone (Co), and presence of new bone due external periosteal reaction (Ne). (magnification: 4x); B-C. New bone observed owed to endosteal reaction (N); presence of many new trabeculae bone (magnification, respectively, 40x and 100x).

The second group (Biomat) underwent surgery for implantation of alloplastic biomaterial (positive control group). As observed in Fig. 2, it presented a ticker bone formation compared to the negative control group, which completely filled the non-critical defect area. It is also possible to see the presence of adipocytes, macrophages, and megakaryocytes (Fig. 1). The remnant biomaterial (partially resorbed) was surrounded by islands of new bone, and zones of bone formation occurred in a centripetal direction (Fig. 1).

In the Zol.BP group, the bone defect was filled with a new bone bridge with a small amount of medullary tissue (Fig. 1). A new bone in the inner part can be observed which was a result of an endosteal reaction; in addition, considerable amounts of newly trabeculae of bone was observed within the medullary tissue (Fig. 1). The same pattern of response was achieved in the group receiving Zol.BP and biomaterial with considerable numbers of new trabeculae being observed (Fig. 1). In addition, the biomaterial was observed as non-completely resorbed, surrounded by new bone in close contact (Fig. 1).

**Figure 3:**
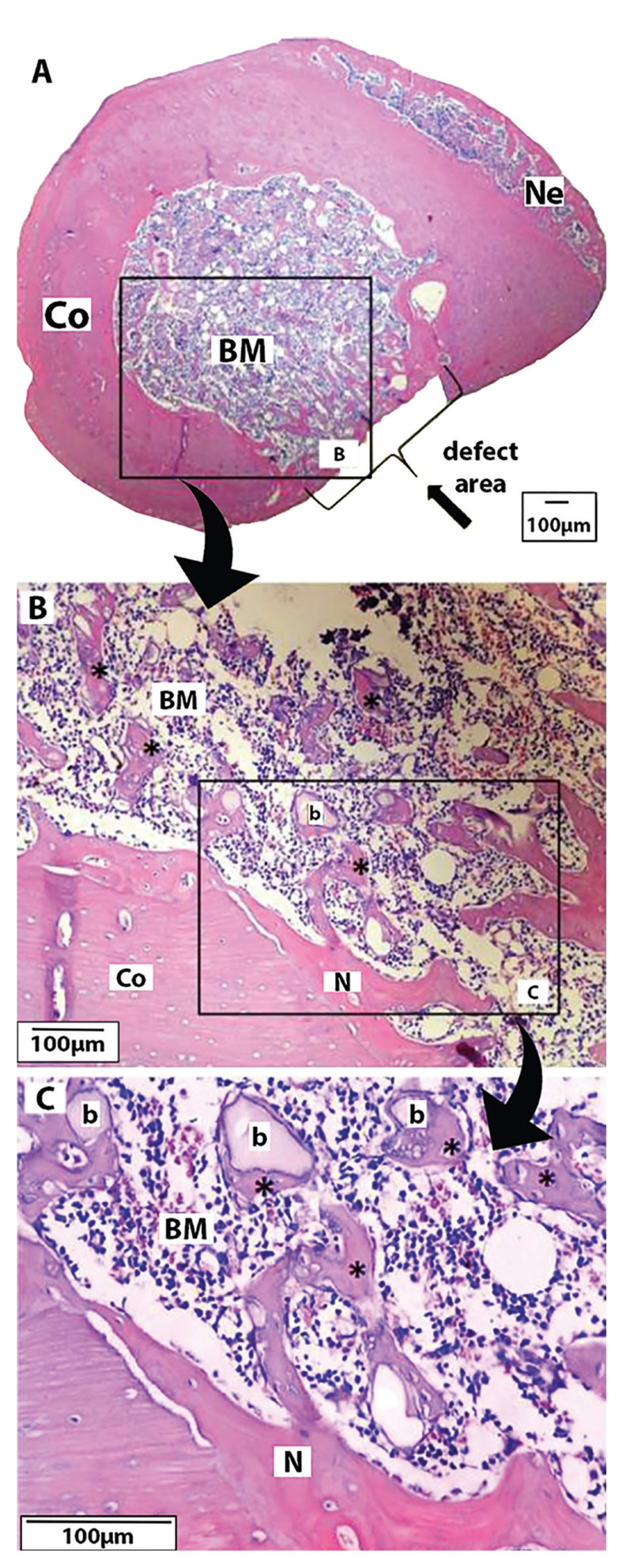
Clo.BP + Biomat Group. A. Defect area filled with a thin mature bone layer (lateral arrow) and presence of periosteal reaction (Ne) (magnification: 4x); B-C. Bone marrow (BM) interspersed with delicate new bone trabeculae (*) in close association with the biomaterial (b); presence of endosteal (N) (magnification, respectively, 40x and 100x).

Similar to previous groups, the Clo.BP group had the defect area filled with mature bone (Fig. 2); it is noTable that its new bone was thicker than in the other groups. Moreover, nearly half of the medullary cavity had new bone trabeculae scattered, encircled by cortical bone (Fig. 2). A small volume of new bone was observed due to endosteal and periosteal (Fig. 2) reaction. A similar pattern to that observed in the Clo.BP group was also observed in the Clo.BP + Biomat group. Otherwise, it had the defect area filled with a thin mature bone, with new bone present throughout the medullary cavity in the form of delicate trabeculae, which were in close association with the biomaterial (juxtaposition) (Fig. 3). In addition, larger particles of biomaterial were found in this group, similar to Zol.BP, compared to the positive control group (Biomat).

**Figure 4:**
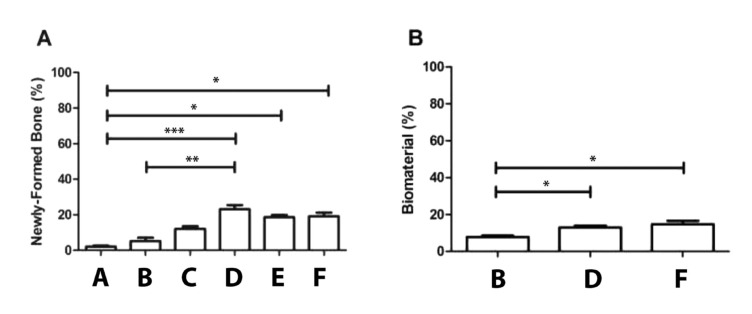
Histomorphometry analysis. A) Quantification of newly formed bone (%) in the control groups (Negative and Biomat) and groups receiving Biomat+Zol.BP (*** *p*<0.001_A-D; ** *p*<0.01_B-D), Clo.BP alone (* *p*<0.05_A-E), and Biomat+Clo.BP (* *p*<0.05_A-F). B) Quantification of remnant biomaterial after 30 days of assay. Note the elevated content of biomaterial in the groups receiving Biomat+Zol.BP (* *p*<0.05) and Biomat+Clo.BP (* *p*<0.05) compared with Biomat alone.

Histomorphometrically, the negative control group (blood clot [A]) had 2% bone formation compared to the positive control (Biomat [B]), which presented 5% newly formed bone. The experimental groups without biomaterials Zol.BP (C) and Clo.BP (E) achieved a respectively mean bone formation 6-fold more (12%, *p*>0.05) and 9.5-fold more (19%, *p*<0.05) than the negative control. In the presence of the biomaterial, Zol.BP + Biomat group (D) presented the highest value found for newly formed bone, with 24% of the area filled out, 12-times more than the negative control (A) (*p*<0.001) and 4.8-fold more than the positive control group (B) (*p*<0.01). This fact shows that the association of zoledronate with alloplastic biomaterial was the most effective for the bone formation process. Clo.BP (E) and Clo.BP + Biomat (F) groups reached the same results for new bone formation (19%), with a statistically significant difference compared to the negative control group (A) (*p*<0.05) (Fig. 4).

Analyzing the volume of remnant biomaterial (Fig. 4), it was observed that the positive control group had 8% of the total area occupied by its presence, whereas the experimental groups, Zol.BP + Biomat (*p*<0.05) and Clo.BP + Biomat achieved 15% (1.87-fold more) (*p*<0.05). There was no statistical difference between the experimental groups treated with biomaterial.

## Discussion

Bone healing depends directly on the balance between bone formation and resorption. Any factors that alter this balance may impact treatment duration, outcome, and prognosis. As BPs inhibit osteoclast activities, it results in decreased bone resorption. It may negatively affect bone remodeling, prolonging the bone healing period. On the other hand, BPs have a positive effect on bone healing in animal studies (15,16). Thus, the purpose of this study was to verify the impact of two different generations of BPs, clodronate (1st generation) and zoledronate (3rd generation), systemically applied, on the bone repair of a non-critical defect made in the rats’ femur, assisted or not by a synthetic biomaterial. The outcomes of this study show that the utilization of first and third generations of BPs favorably impacted bone healing on non-critical defects, mainly in the Zol.BP + Biomat group.

BPs bind to bone minerals and inhibit the dissolution of hydroxyapatite (HAp). The literature shows that zoledronic acid has a good binding ability to HAp (17) and a stronger ability to inhibit farnesyl pyrophosphate synthase (FPPS), resulting in stronger osteoclast inhibition. This potential was observed in our experimental groups, where the biomaterial in the BPs with biomaterial groups had a significantly lower degradation. Previous articles explored the impact of BPs on bone formation when associated or not with bone graft materials (9-11,18). The utilization of BPs applied systemically or locally to treat bone defects in association with an alloplastic biomaterial showed no recognizable changes in osteogenesis (18). In such a manner, the quantitative and qualitative evaluations performed in our study have shown that zoledronate and clodronate act differently, and both had a significant impact on osteogenesis, which was statistically observed compared to the control groups.

Clodronate (first generation of BP) has the advantage of not inducing MRONJ, even at high concentrations (19), and has already been associated with the development of biomaterials. A study (20) successfully demonstrated the incorporation of clodronate to a layer of the biomimetic structure of calcium phosphate (Ca-P) biomaterial; another study demonstrated the incorporation of clodronate to bioglass bone graft *in vitro*, which created a favorable environment for an increased bone formation (21). However, this present study is the first to report an *in vivo* assessment of the concomitant use of clodronate and alloplastic biomaterial. Interestingly, in this study, clodronate presented no difference when associated with the use of the biomaterial, but its impact on bone formation, even in the absence of biomaterial, was strongly evident in the qualitative and quantitative analysis. Otherwise, it was clear that in the presence of clodronate and zoledronate, the synthetic biomaterial demonstrated its osteoconductive property since most of the newly formed bone surrounded the particles of biomaterial in juxtaposition.

Zoledronate potentiates the effects of the biomaterial used, presenting the highest impact on bone formation compared to other groups. Otherwise, clodronate alone or with the biomaterial was able to increase bone formation, even though the newly formed bone showed more dispersed and strongly associated with the biomaterial. Some authors (22) investigated the effect of zoledronate systemically administered in the incorporation of bioglass; the results showed an effective process for the new bone formation. Another study (23) used alendronate (second generation of BPs) systemically administered via subcutaneous injections (daily for 12 weeks); the outcome demonstrated there was stimulation of the bone formation, which was associated with autogenous bone graft in a rat model. These studies corroborate our results, demonstrating that nitrogenous BPs had a beneficial result in bone formation when associated with bone grafts.

Therefore, it is still unclear whether bisphosphonate therapy actually indirectly affects bone healing when associated with bone grafts. Some animal studies demonstrated that bisphosphonate used after surgery could increase bone formation and delay remodeling (24-26). In a study involving high tibial osteotomy, the authors (27) reported that the infusion of zoledronate did not affect bone healing. Otherwise, in the present study, BPs of the first and third generations positively affected bone healing and seemed to slow down the resorption of the biomaterial since, in the groups without the drug, the biomaterial fragments were smaller, as evidenced by both qualitative and quantitative analysis. The present results do not provide a reason for this decrease in resorption, but this fact may be related to the direct and indirect effects of BPs in osteoclastogenesis.

Even though our results were favorable for the experimental groups proposed, it must be careful to use BPs due to the possibility of jaw osteonecrosis. Notably, in this study, the zoledronate dose employed corresponds to previously reported concentrations unrelated to the experience of osteonecrosis (19), thus avoiding the risk of negative effects. Therefore, its administration creates the possibility of moderate side effects (myalgia, arthralgia, and fever) or severe side effects (avascular bone necrosis, cardiac arrhythmia, impairment of renal function, hypocalcemia, delayed bone healing, and primary death) (28).

## Conclusions

It was possible to conclude that the systemic BP's positive influence and impact on the newly formed bone happened. Clodronate (1st generation of BP) had a high osteogenic potential, even in the absence of the biomaterial, whereas zoledronate (3rd generation of BP) had an impact on the new bone formation only when associated with the alloplastic biomaterial.

## Figures and Tables

**Table 1:** Distribution of animals and groups according to the treatment.

Nº of days	(A) Negative Control Group	(B) Positive Control (Biomat) Group	(C) Zol.BP	(D) Biomat+Zol.BP	(E) Clo.BP	(F) Biomat+Clo.BP
0 day	-	-	Intraperitoneal application of zoledronic acid (every 30 days)	Intraperitoneal application of zoledronic acid (every 30 days)	Intraperitoneal application of clodronate (every 30 days)	Intraperitoneal application of clodronate (every 30 days)
30 days	-	-				
60 days	Surgery Blood clot	Surgery Biomat Group	Surgery (Zol.BP + blood clot)	Surgery (Biomat + Zol.BP)	Surgery (clodronate + blood clot)	Surgery (Biomat + clodronate)
90 days	Euthanasia	Euthanasia	Euthanasia	Euthanasia	Euthanasia	Euthanasia

Zol.BP = zoledronic acid (Bisphosphonates); Biomat = biomaterial.
